# Telemedicine Utilization in Tertiary, Specialized, and Secondary Hospitals in Thailand

**DOI:** 10.1089/tmr.2024.0027

**Published:** 2024-08-05

**Authors:** Piyada Gaewkhiew, Nitichen; Kittiratchakool, Chotika Suwanpanich, Thanayut Saeraneesopon, Thanakit Athibodee, Suthasinee Kumluang, Tanainan Chuanchaiyakul, Sichen Liu, Saranya Chanpanitkitchot, Arthit Laosuangkul, Wanrudee Isaranuwatchai

**Affiliations:** ^1^ Health Intervention and Technology Assessment Program (HITAP), Nonthaburi, Thailand.; ^2^ Department of Community Dentistry, Faculty of Dentistry, Mahidol University, Bangkok, Thailand.; ^3^ Department of Obstetrics and Gynecology, Rajavithi Hospital, College of Medicine, Rangsit University, Bangkok, Thailand.; ^4^ Department of Mental Health, Suansaranrom Psychiatric Hospital, Ministry of Public Health, Suratthani, Thailand.; ^5^ Institute of Health Policy, Management and Evaluation, University of Toronto, Toronto, Canada.

**Keywords:** telemedicine, telehealth, utilization, data analytics, hospital

## Abstract

**Introduction:**

COVID-19 has accelerated the adoption of telemedicine for counseling, follow-up examination, and treatment purposes. The official guidelines in Thailand were launched to regulate or frame the protocols for health care professions and teams in different organizations.

**Objectives:**

To explore the trend of telemedicine utilization in selected hospitals in Thailand and to understand the characteristics of patients who used telemedicine from 2020 to 2023.

**Methods:**

This retrospective secondary data analysis was conducted in four hospitals in Thailand: two tertiary care (T1 and T2) hospitals, one secondary care (SN) hospital, and one specialized (SP) hospital. Data were routinely collected when services were provided and were categorized into telemedicine outpatient department (OPD) visits or onsite OPD visits. The data included demographic information (age, sex), date and year of service, location (province and health region), and primary diagnosis (using International Statistical Classification of Diseases and Related Health Problems 10th Revision codes). Descriptive analysis was conducted using R and STATA software.

**Results:**

All four hospitals reported an increase in telemedicine use from 2020 to 2023. The majority of telemedicine users were female (>65%) at all hospitals except for the SP hospital (44%). Participants aged 25–59 years reported greater utilization of telemedicine than did the other age-groups. The within-hospital comparison between OPD visits before and after telemedicine was significant (*p* < 0.001).

**Conclusion:**

The situation during the COVID-19 pandemic and the transition to the post-COVID-19 era impacted telemedicine utilization, which could support national monitoring and evaluation policies. However, further studies are needed to explore other aspects, including changes in telemedicine utilization over time for longer timeframes, effectiveness of telemedicine, and consumer satisfaction.

## Introduction

Telemedicine is defined as “the delivery of healthcare services where distance is a critical factor by all healthcare professionals using information and communication technologies for the exchange of valid information for diagnosis, treatment and prevention of disease and injuries in the interest of advancing the health of individuals and their communities,”^1,2^ which could help to increase access to health care services and enhance health care delivery.^3^ However, prior to the coronavirus disease (COVID-19) pandemic, there was slow progress in adopting telemedicine in practice, especially in developing countries where the technology was unavailable or inconvenient.^4^ COVID-19 accelerated the acceptance of telemedicine utilization in both medical providers and patients.^5^ In addition, the World Health Organization has provided recommendations on digital interventions for health system strengthening,^6^ which include examples of interventions such as monitoring procedures between patients and physicians and education and training tools among health care workers. These interventions could reduce the limitation of resources and improve access to health care in the population,^7^ especially among those who live in rural areas or who are experiencing a barrier to health care utilization. In addition, a previous review of telemedicine in Southeast Asia region in 2023 revealed that only a few countries, including Singapore,^8^ Malaysia,^9^ Vietnam,^10^ Philippines,^11^ and Thailand,^12^ have guidelines for telemedicine utilization but not in detail, especially regarding training in telemedicine usage^6^; moreover, an e-health foundation is needed for infrastructure development to enable telemedicine and e-health services.^13^

During the COVID-19 pandemic, COVID-19 accelerated the use of telemedicine instead of onsite hospital visits, as did the National Health Security Office (NHSO) policy, which encouraged hospitals and health care professionals to provide telemedicine services.^14,15^ Similarly, in Thailand, the use of mobile technology or telemedicine for consulting was a concern in terms of implementation during the prepandemic period due to a lack of supportive systems and technical expertise.^16^ Then, COVID-19 was first detected in Thailand in the first trimester of 2020. The Thai government implemented lockdown measures, including limiting travel across the country and restricting group gatherings. Additionally, during the COVID-19 pandemic, the population had to practice physical distancing in general as well as in health care services. In-person health care services were allowed only for essential cases.^17^ Telemedicine was mostly adopted for symptom screening to confirm the risk of COVID-19, and follow-up examinations, as social distancing measures, were used to quarantine individuals with COVID-19.^18,19^

Additionally, telemedicine was utilized for specific procedures, such as lab result notifications, physical examinations, and consulting services.^20^ Moreover, some private hospitals provided telemedicine for follow-up visits or consultation services in light of social distancing measures and the necessity of hospital visits. The NHSO reported on telemedicine utilization via mobile phones used to contact patients for screening, follow-up communication, and emergency notifications. Additionally, in 2021, the NHSO launched a policy related to telemedicine, and telemedicine was encouraged to improve communication, assist elderly individuals, and strengthen health care services, which officially started in January 2023. After the implementation of telemedicine, guidelines related to telemedicine were launched via official organizations, including the Department of Medical Services, the Thai Medical Council, and the Thai Traditional Medical Council.^12,21,22^

Due to the unprecedented situation in Thailand, hospitals have launched policies related to telemedicine, such as screening, follow-up examination, reporting of laboratory results, and procedures as treatment services.^23–25^ The Ministry of Public Health (MoPH) also encouraged telemedicine among health care professionals to reduce the risk of COVID-19 and the number of onsite visits. In addition, the NHSO in Thailand reported that the average number of outpatient visits in the capital area was lower than that in other parts of the country.^26^ Given that telemedicine is still a novel alternative for both health care professionals and patients in Thailand, few reports have investigated the trends and patterns of telemedicine utilization at multicenters or the characteristics of users. A previous study in Thailand during COVID-19 reported that telemedicine utilization was mostly correlated with COVID-19 new cases detected, which also suggested to explore the pattern of using telemedicine in Thailand.^27^ These findings could support the monitoring of health care services as well as the evaluation of telemedicine programs at the national level. Thus, this study aimed to explore the trend of telemedicine utilization among patients in specific hospitals in Thailand and to better understand the characteristics of patients who used telemedicine in Thailand.

## Materials and Methods

### Study overview and data sources

**FIG. 1.**
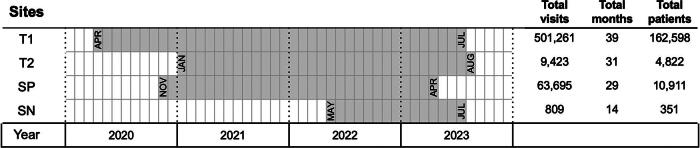
Available extracted data from study hospitals.

This retrospective secondary data analysis was conducted in four hospitals (coded as T1, T2, SP, and SN) that most frequently recorded telemedicine services provided in Thailand.^18^ These four hospitals are two tertiary care (T1 and T2) hospitals, one secondary care (SN) hospital, and one specialized (SP) hospital.^28^ The T1, T2, and SP hospitals provide tertiary care, and the SN hospital provides secondary care. In Thailand, hospitals are categorized by care level and facilities, including numbers of beds. Specifically, the T2, SP, and SN hospitals are under the MoPH, but the T1 hospital is under the Ministry of Higher Education, Science, Research, and Innovation. The capacity of each hospital is different: T1 hospital has 1,023 beds, T2 hospital has 1,200 beds, SP hospital has 480 beds, and SN hospital has 215 beds (Figure 1).

Data are routinely collected when services are provided and are categorized into telemedicine outpatient department (OPD) visits and onsite OPD visits by clinics. The data were retrieved from hospital records in the Hospital Information System from 2020 to 2023 and arranged according to the different time series of each hospital for pre-telemedicine and post-telemedicine OPD onsite visits. Specifically, the T1 hospital provided telemedicine services from April 2020 until July 2023; the T2 hospital, from January 2021 to August 2023; the SP hospital, from November 2020 to April 2023; and the SN hospital, from May 2022 to July 2023.

### Variables

The variables of interest were extracted, including demographic information (age, sex), date of service, location (province, health region, mode), primary diagnosis, or PDX, which were grouped following the International Statistical Classification of Diseases and Related Health Problems 10th Revision (ICD-10).^16^ Age was grouped as 0–5 (preschoolers), 6–24 (children and adolescents), 25–59 (working adults), and 60 years or older (older adults).^29^ All the data were anonymized, and there was no direct contact or communication with the patients.

### Health care service utilization

**FIG. 2.**
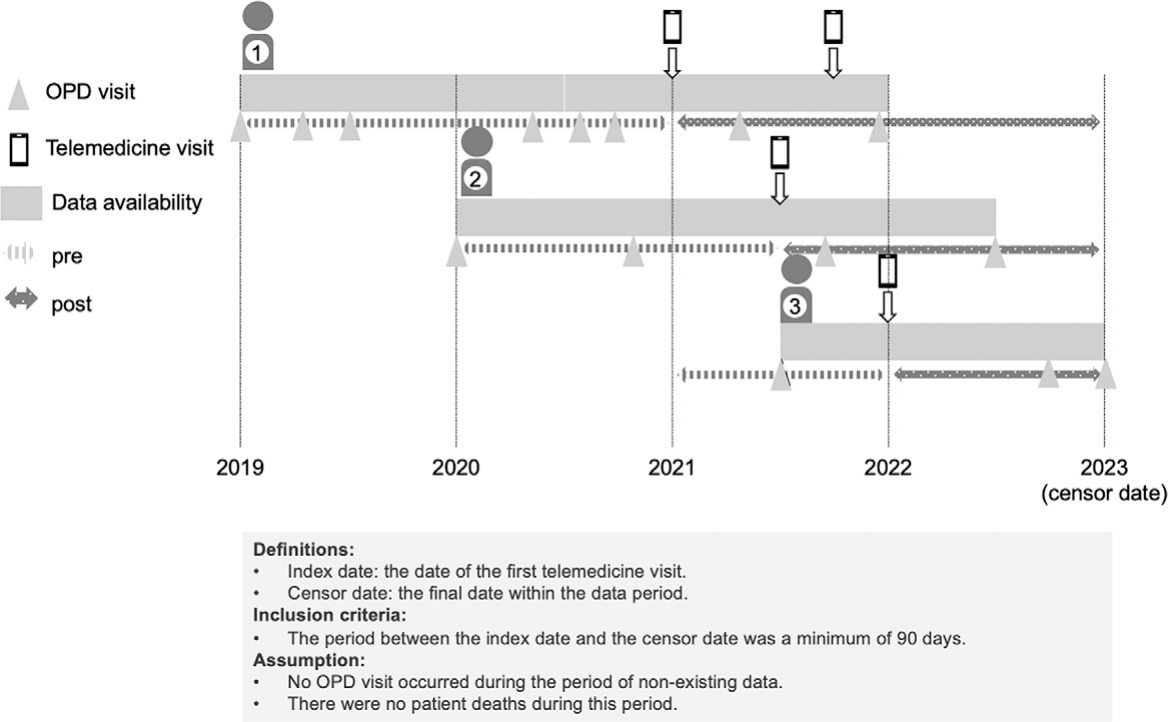
Timeframe for calculating pre-telemedicine and post-telemedicine periods.

In this study, health care service utilization was considered both onsite and via telemedicine in the OPD. The “index date” was defined as the first day of telemedicine use. Preceding this index date, any OPD onsite visits were categorized as “pre-telemedicine OPD onsite visits.” After the index date, the OPD onsite visits were referred to as “post-telemedicine” visits. To be included in this study, patients had to meet specific criteria: the duration between the “index date” and the “censor date” (used as the cutoff point) had to exceed 90 days to exclude participants who had too short duration after first use of telemedicine; for example, ID01 had only 1 day after first use of telemedicine as post-telemedicine duration, which is unable to analyze onsite OPD visit during the post-telemedicine period. Patients with a duration between the “censor date” and the first telemedicine visit of <90 days were excluded from the analysis. In the comparison between the duration of the pre-telemedicine period and the duration of the post-telemedicine period, the shorter of these two periods was defined as the “timeframe.” This timeframe was then utilized to divide the pre-telemedicine and post-telemedicine periods equally, as shown in Figure 2.

### Statistical analysis plan

A descriptive analysis was used to describe the demographic data (age, sex), health conditions (primary diagnosis), and service utilization (number of OPD visits) of patients who used telemedicine. Age-groups were grouped following the NHSO age-group categories.^30^ The ICD-10 (22 chapters) and 5 specific chapters related to mental and behavioral disorders were used. The comparison of the mean values of the OPD onsite visits between pre-telemedicine and post-telemedicine visits was performed via paired *t*-tests. All the statistical analyses were conducted via R software version 4.2.2 and STATA version 16.0.

This study was approved by the ethics committees of all the study sites (document number: COA. MURA2023/488; 067/2023; SSR.REC 004/2566; COA No. IHRP2023042).

## Results

The duration of available data from the four different hospitals differed: the T1 hospital had 39 months, the T2 hospital had 31 months, the SN hospital had 14 months, and the SP hospital had 29 months of data (Figure 1).

The overall numbers of telemedicine visits at the T1, T2, SN, and SP hospitals are shown in Table 1. Among the total telemedicine visits, the T1 hospital had the greatest number of telemedicine visits, with 501,261 visits; the SP hospital had 63,695 visits; the T2 hospital had 9,549 visits; and the SN hospital had 758 visits.

**Table 1.** Telemedicine Patients’ Characteristics Among Four Hospitals in Thailand (*N* = 575,263)

Characteristics	T1	T2	SN	SP
Duration (months)	39	31	14	29
Total number of visits	501,261	9,549	758	63,695
Total patients	170,004	4,822	338	10,911
Age				
Mean (years)	53.4	46.5	58.73	50.15
[Min–max; SD]	[0–109; 21.2]	[0–97;14.3]	[9–93; 14.15]	[16–103; 16.98]
Age-group (*n* [%])				
0–5 years	4,568 [2.69]	42 [0.87]	0 [0]	0 [0]
6–24 years	13,032 [7.67]	422 [8.75]	6 [1.78]	441 [4]
25–59 years	74,526 [43.84]	3,496 [72.5]	172 [50.89]	7,238 [66]
60+ years	77,878 [45.81]	862 [17.88]	160 [47.34]	3,232 [30]
Sex (*n* [%])				
Male	56,480 [33.22]	196 [4.06]	99 [29.29]	6,042 [55.38]
Female	113,523 [66.78]	4,626 [95.94]	239 [70.71]	4,869 [44.62]
Number of visit(s) (*n* [%])				
1 time	76,659 [45.09]	3,029 [62.82]	123 [36.39]	1,319 [12.09]
2 times	33,886 [19.93]	1,112 [23.06]	78 [23.08]	1,154 [10.58]
3 times	18,360 [10.80]	333 [6.91]	94 [27.81]	1,269 [11.63]
4 times	11,386 [6.70]	146 [3.03]	28 [8.28]	1,373 [12.58]
5 times and above	29,713 [18]	202 [4.19]	15 [4.44]	5,796 [53.12]

SD, standard deviation.

Regarding age-group, telemedicine was mostly used by the 25–59 age-group, except at the T1 hospital, where the 60 and older age-group mostly used telemedicine. The mean age of the telemedicine patients from all four hospitals ranged between 47 and 60 years. Females were more likely to use telemedicine in the T1, T2, and SN hospitals than males, whereas males used telemedicine more often in the SP hospital. Regarding the number of visits per patient, onetime telemedicine users were the most common in the T1, T2, and SN hospitals.

Table 2 presents the top five primary diagnoses in each hospital, categorized by ICD-10 codes. In the T1 hospital, essential (primary) hypertension (I10) was the most common diagnosis, accounting for 6.2% of the patients (26,479 patients). Moreover, the SN hospital also recorded essential (primary) hypertension as the most common diagnosis, with 40.90% of patients (310 patients) being affected by this condition.

**Table 2.** The Top Five Primary Diagnoses Among Telemedicine Patients in Four Hospitals

	T1			T2			SP			SN		
	ICD-10^22^	Number of visits	[%]	ICD-10^22^	Number of visits	[%]	ICD-10^22^	Number of visits	[%]	ICD-10^22^	Number of visits	[%]
Top 1	I10	26,479	[6.20]	Z099	2,572	[27.77]	F2003	6,117	[9.60]	I10	310	[40.90]
Top 2	F322	14,569	[3.41]	H903	1,159	[12.51]	F2000	4,466	[7.0]	E119	251	[33.11]
Top 3	E788	9,256	[2.17]	Z014	1,036	[11.18]	F321	2,796	[4.39]	F322	31	[4.09]
Top 4	E119	7,349	[1.72]	C539	249	[2.69]	F2002	2,730	[4.29]	E789	27	[3.56]
Top 5	I251	6,831	[1.60]	Z505	195	[2.11]	F412	2,588	[4.06]	Z518	15	[1.98]

ICD-10 codes: C539 (malignant neoplasm of cervix uteri, unspecified), E119 (noninsulin-dependent type 2 diabetes mellitus at without complications), E788 (other disorders of lipoprotein metabolism), F412 (mixed anxiety and depressive disorder), F2000 (paranoid schizophrenia, continuous) (including treatment resistant), E788 (other disorders of lipoprotein metabolism), E789 (disorder of lipoprotein metabolism, unspecified), F2002 (paranoid schizophrenia, episodic with stable deficit), F2003 (paranoid schizophrenia, episodic remittent), F321 (moderate depressive episode), F322 (severe depressive episode without psychotic symptoms), H903 (sensorineural hearing loss, bilateral), I10 (essential [primary] hypertension), I251 (atherosclerotic heart disease), Z099 (follow-up examination after unspecified treatment for other conditions), Z014 (gynecological examination [general] [routine]), Z505 (speech therapy), and Z518 (other specified medical care).

ICD-10, International Classification of Diseases and Related Health Problems 10th Revision; SN, secondary care hospital; SP, specialized hospital; T1, tertiary care hospital 1; T2, tertiary care hospital.

However, in the T2 hospital, there was a different profile of primary diagnoses, with follow-up examination after unspecified treatment for other conditions (Z099) and paranoid schizophrenia and episodic remittent schizophrenia (F2003) being the most prevalent conditions, affecting 27.77% (2,572 patients) and 9.6% (6,117 patients), respectively.

In the T1 hospital, the following primary diagnoses were also notable: severe depressive episode without psychotic symptoms, other disorders of lipoprotein metabolism, noninsulin-dependent type 2 diabetes mellitus without complications, and atherosclerotic heart disease.

For the T2 hospital, sensorineural hearing loss (bilateral), gynecological examination (general) (routine), malignant neoplasm of the cervix uteri (unspecified), and speech therapy were among the top primary diagnoses.

In the case of the SP hospital, which is a specialized hospital in mental health, paranoid schizophrenia, continuous depression (including treatment-resistant depression), moderate depressive episode, paranoid schizophrenia, episodic schizophrenia with a stable deficit, and mixed anxiety and depressive disorder were the prominent primary diagnoses.

### Trend of telemedicine utilization over time

**FIG. 3.**
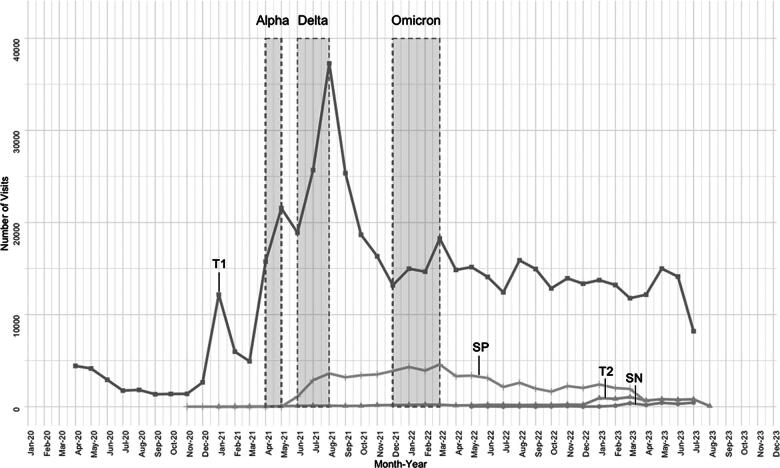
Trend of telemedicine utilization overtime from four hospitals.

The utilization of telemedicine services exhibited varying peaks across hospitals from January 2020 to July 2023. The T1 hospital experienced its highest level of telemedicine utilization in August 2021, coinciding with the surge of COVID-19 cases attributed to the Delta variant. On the contrary, the SP hospital observed its peak utilization in May 2022, aligning with the Omicron wave of the pandemic. The trend in telemedicine utilization dropped in 2023 compared with that in 2021 and 2022; however, there were fluctuations in the transitions between 2022 and 2023. Additionally, Figure 3 shows the comparison among all four selected study hospitals. There was a greater utilization of telemedicine in the T1 hospital than in the T2, SP, and SN hospitals during all periods.

The trends in telemedicine utilization among the four hospitals are shown in Figure 3. The T1 hospital exhibited a peak in terms of telemedicine utilization in August 2021 during the Delta wave of COVID-19 in Thailand. The SP hospital had the highest number of telemedicine utilizations during the Omicron wave (December 2021 to March 2022). The T2 and SN hospitals had lower rates of telemedicine utilization than the T1 and SP hospitals.

In Table 3, the changes in onsite OPD visit utilization during the pre- and post-telemedicine periods are reported, and the data are reported as average 3-month intervals or trimesters. Only 174,336 participants who had sufficient data 3 months after telemedicine utilization were included in this analysis. Across all four hospitals, the average number of regular OPD visits per trimester decreased, except for the T1 hospital, where the average number of visits increased slightly from 1.7 (min: 0, max: 55.9) to 1.9 visits (min: 0, max: 60.5) per trimester. At the T2 hospital, the average number of pre-telemedicine onsite OPD visits was 3.46 per (min: 0, max: 52.5) trimester, which subsequently dropped to 2.69 visits per trimester (min: 0, max: 66.52) after the first use of telemedicine. For the SN hospital, the average number of pre-telemedicine onsite OPD visits was 2.13 per trimester (min: 0, max: 11.08), and this number decreased to 1.07 visits per trimester (min: 0, max: 9.49) after the initiation of telemedicine services. Finally, the SP hospital reported a pre-telemedicine average of 1.15 onsite OPD visits per trimester (min: 0, max: 19.1), which decreased to 0.61 visits per trimester (min: 0, max: 11.5) following the adoption of telemedicine. The difference in the mean number of OPD onsite visits before and after telemedicine at each hospital was statistically significant (*p* value <0.001).

**Table 3.** The Pre-Post Telemedicine Utilization per Trimester in the Outpatient Department Among Four Hospitals (*N* = 174,336)

Hospital	T1	T2	SN	SP
OPD				
Total number of patient (person)	159,877	3,657	257	10,545
Pre-telemedicine (number of visits)				
Mean [SD] per trimester	1.7 [1.9]	3.46 [4.61]	2.13 [1.48]	1.15 [0.876]
Min	0	0	0	0
Max	55.9	52.5	11.08	19.1
Post-telemedicine (number of visits)				
Mean [SD] per trimester	1.9 [2.4]	2.69 [4.13]	1.07 [1.21]	0.610 [0.609]
Min	0	0	0	0
Max	60.5	66.52	9.49	11.5

OPD, outpatient department.

**FIG. 4.**
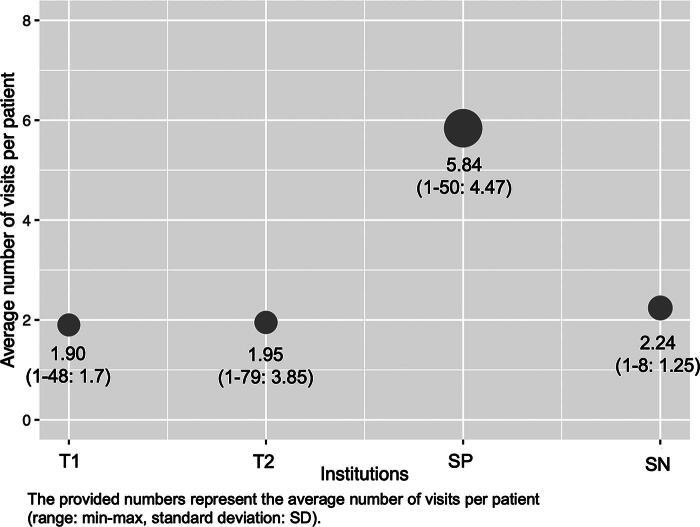
The average of telemedicine utilization overtime per patient and number of patients who used telemedicine by frequency from four hospitals.

The frequency of telemedicine utilization over the course of the study period and patient grouping by frequency are shown in Figure 4. The majority of users in the T1, T2, and SN hospitals were 1-time telemedicine users (45%, 63%, and 36%, respectively), whereas the majority of users (53%) in the SP hospital used telemedicine services at least five times.

## Discussion

The key findings of this study revealed the patterns of telemedicine utilization and its effects on onsite OPD visits across four hospitals in Thailand. Telemedicine adoption varied by age-group and sex across the four hospitals. The female and working age-groups (25–59 years old) used telemedicine more than did the male and other age-groups. Interestingly, females were the predominant telemedicine users in this study, which is consistent with the findings from healthcare utilization studies in the United States that show females have more telemedicine visits than males, supported by greater time availability^31^ and health-seeking behaviors.^32,33^ Essential hypertension was a common primary diagnosis in the T1 and SN hospitals, whereas the T2 hospital had distinct primary diagnoses, such as follow-up examinations and paranoid schizophrenia. Paranoid schizophrenia and episodic remittent schizophrenia were the most common primary diagnoses in the SP hospital. This variation could be the result of differences in telemedicine policies and implementation across hospitals. Telemedicine utilization peaked at different times across hospitals, aligning with waves of COVID-19, and demonstrated some fluctuations from January to May 2023.^34^ Additionally, although not significantly so, onsite OPD visits declined in all hospitals, with the exception of the T1 hospital, which reported a slight increase. These findings emphasize the dynamic nature of telemedicine adoption and its influence on traditional health care service utilization.^35^

Our findings show that telemedicine has been increasingly used since the beginning of the COVID-19 pandemic or January 2020 and has remained an integral part of medical care up to the present, similar to other countries.^36^ Additionally, a review of telemedicine utilization revealed that, compared with before January 2020, the utilization of health care services increased in the United States, the United Kingdom, Canada, China, and Africa during the COVID-19 pandemic.^37,38^ As telemedicine provides more convenient services than in-person visits, the study hospitals used telemedicine for follow-up visits or for lab result reports, which could minimize indirect costs for patients.^39^ However, the barriers to accessing telemedicine are regulatory, legal, and reimbursement systems.^40^ In addition, dissatisfaction during interactions with doctors and poor connectivity to telecommunication networks have been reported as reasons for the discontinuation of telemedicine utilization among patients and health care professionals.^41^ These barriers to telemedicine should be considered in the future not only for research but also for policies aimed at determining the importance of monitoring and evaluating the continuation of telemedicine programs.

In this study, real-world hospital datasets were compiled from diverse hospitals in Thailand to provide an overview of telemedicine utilization in the Thai population. The strength of this study was the reporting of the general characteristics of telemedicine users, including demographic characteristics and primary diagnoses. In addition, the number of OPD visits was recorded before and after the first use of telemedicine, which could help in exploring the trends of telemedicine utilization over time in Thailand. Some hospitals implemented telemedicine before the NHSO and the MoPH launched this policy. Furthermore, primary diagnoses could help identify specific health care needs related to different diseases, as indicated by the diverse use of telemedicine at each hospital. For example, the T2 hospital implemented telemedicine in specific clinics, including gynecological and otorhinolaryngology (ear, nose, and throat: ENT) clinics. The most frequently reported primary diagnoses for patients from the T2 hospital were diseases related to gynecological examination and speech training. The hospital data could reflect hospital policy related to telemedicine services in each focus.

## Limitations

This study has several limitations that need to be addressed. Due to the limited data on certain variables, such as duration of visits and cost of procedures, the determinants of telemedicine use could not be explored well. An advanced statistical analysis was not performed because of the availability of data frames and the differences in the data between each hospital. Additionally, the implementation of telemedicine, including monitoring and evaluation, still needs to be investigated. Thus, these findings should be interpreted with caution, as the adjustment of the analysis was limited.

## Recommendation

The centrally predetermined regulation of telemedicine services could solidify the implementation by which, as an example, Singapore’s health regulation group constructed concrete guidelines for telemedicine service delivery, serving as a backbone for the national health system service.^42^ Other countries in Southeast Asia also have telemedicine guidelines with different approaches and purposes for each procedure, such as short message services, fax, chats on various platforms, or audio via telephone. These points could benefit future research by allowing exploration and evidence-based health care policy to develop procedures as well as the protocol and guideline in telemedicine for better service and clinical outcomes.

## Conclusion

This study showed the characteristics and primary diagnoses of telemedicine users to support the monitoring and evaluation of current telemedicine policy in Thailand. Due to the lack of evidence on telemedicine utilization in Thailand, further exploration is needed to determine the factors contributing to these trends in utilization and clinical profiles between users and nonusers. However, this study could help understanding the foundational stage of telemedicine in such regions, which is crucial as it provides insights into the barriers and opportunities in developing countries, which could help other countries that also face the similar context. In addition, the broader implications for health care delivery via telemedicine as well as the development of central guidelines for telemedicine utilization should be addressed in the future.

## Abbreviations Used

COVID-19: coronavirus disease
ICD-10: International Classification of Diseases and Related Health Problems 10th Revision
MoPH: Ministry of Public Health
OPD: outpatient department
SD: standard deviation
SN: secondary care hospital
SP: specialized hospital
T1: tertiary care hospital 1
T2: tertiary care hospital
